# Transcriptome and Metabonomics Analysis Revealed the Molecular Mechanism of Differential Metabolite Production of *Dendrobium nobile* Under Different Epiphytic Patterns

**DOI:** 10.3389/fpls.2022.868472

**Published:** 2022-05-17

**Authors:** Qingqing Li, Chaobo Liu, Ceyin Huang, Mufei Wang, Teng Long, Jingyi Liu, Junhua Shi, Junli Shi, Lin Li, Yuqi He, De-Lin Xu

**Affiliations:** ^1^Department of Medical Cell Biology, Zunyi Medical University, Zunyi, China; ^2^Affiliated Hospital of Zunyi Medical University, Zunyi, China; ^3^School of Medicine, Zunyi Medical University, Zunyi, China

**Keywords:** *Dendrobium nobile* (Shihu), epiphytic patterns, metabonomic, transcriptome, molecular mechanism

## Abstract

The cultivation medium of *Dendrobium nobile* has an effect on the contents of its main medicinal components, but the specific mechanism is still unclear. In this study, the callus, seedlings, rhizomes, and leaves of *D. nobile* were sequenced for the PacBio SMRT. The 2-year-old stems were selected for the Illumina sequencing and metabolome sequencing to analyze the genetic mechanism of metabolic differences under different epiphytic patterns. As a result, a total of 387 differential genes were obtained, corresponding to 66 differential metabolites. Different epiphytic patterns can induce a series of metabolic changes at the metabolome and transcriptome levels of *D. nobile*, including flavonoid metabolism, purine metabolism, terpenoid backbone biosynthesis, amino acid metabolism, and alpha-linolenic acid metabolic, and related regulatory genes include *ALDH2B7, ADC, EPSPS-1, SHKA, DHAPS-1, GES, ACS1, SAHH, ACS2, CHLP, LOX2, LOX2.3*, and *CYP74B2*. The results showed that the genetic mechanism of *D. nobile* under various epiphytic patterns was different. In theory, the content of metabolites under the epiphytic patterns of Danxia stone is higher, which is more suitable for field cultivation.

## Introduction

As a perennial herb of *Dendrobium* of Orchidaceae, *Dendrobium nobile* Lindl. contains alkaloids, terpenoids, phenols, flavonoids, dibenzyl, and glycosides with a high medicinal value (Ling-Hu et al., 2021). At present, sesquiterpenes (Wang et al., 2020), phenanthrene (Cheng et al., 2020), dibenzyl derivatives (Zhang et al., 2019), phenanthraquinone (Liu Y. H. et al., 2021), and other compounds have been isolated. Among them, *D. nobile* alkaloid (ADNL) was conducive to the expression of liver glucose and lipid metabolism genes, played a regulatory role in metabolic disorders (Xu et al., 2017), had a neuroprotective effect, and can cure Alzheimer’s disease (Li et al., 2022). *D. nobile* polysaccharide had an antiviral effect and had broad development prospects (Li Z. et al., 2020). *D. nobile* sesquiterpenes had physiological activities in neuroprotection, immune regulation, and antitumor (Ma et al., 2019). Among the *Dendrobium* recorded in the Pharmacopoeia of the People’s Republic of China, only *D. nobile* has the ability of treating weakness of the five internal organs, strengthening Yin and benefiting essence, holding thick stomach for a long time, and tonifying the kidney and benefiting power, and the alkaloid content in *D. nobile* was the highest among all herbs of *Dendrobium*, which was the main source of medicinal *Dendrobium* (Wang, 2021). The extract of *D. nobile* can also improve constipation (Wang et al., 2021), and the color of the flower was gorgeous, which had an ornamental value. It had moisturizing, whitening, antiaging, and other effects (Nie et al., 2020), and had good application prospects in the cosmetics industry (Yu et al., 2020). It was an economic plant with medicinal and ornamental value.

The quality of Chinese medicinal materials was affected by cultivation methods and environmental conditions, resulting in different synthesis and accumulation of secondary metabolites, and different types and contents of secondary metabolites are responsible for the differences in the quality of Chinese medicinal materials (Guo et al., 2020). The results of the study by Li Y. et al. (2020) showed that the cultivation area, cultivation substrate, and soil conditions had effects on the quality of wendangshen (Jiao et al., 2021), the accumulation of various components in *Phellinus igniarius* (Li, 2020), the yield and quality of *Paeonia lactiflora* Pall (Meng et al., 2020), and so on. Therefore, to ensure the quality of traditional Chinese medicine, the conditions suitable for the growth of traditional Chinese medicine can be selected, and the growth and secondary metabolism of traditional Chinese medicine can be balanced. *D. nobile* was mainly distributed in Guizhou, Shaanxi, Sichuan, and other places in China. The quality of Chishui and Xishui in Guizhou was the best. Among them, the planting technology of Chishui in Guizhou was the most advanced, mainly cultivated on Danxia stones. The cultivation medium of *D. nobile* had an impact on the content of its main medicinal components (Zhang J. Q. et al., 2020). Therefore, it is of great significance to study the effects of different epiphytic patterns on the secondary metabolites of *D. nobile*.

In recent years, with the development of science and technology, transcriptome sequencing technology (RNA-Seq) (Liu H. et al., 2021) and other high-throughput sequencing technologies are more widely used, mainly for functional gene mining (Shovlin and Tropea, 2018), gene network analysis (Pratapa et al., 2020), genetic mechanism analysis (Xu et al., 2020), and so on. The combination of transcriptome and metabolome analysis can more comprehensively analyze the regulatory mechanism of secondary metabolite biosynthesis (Mao et al., 2021). At present, the transcriptome of *D. nobile* has been studied, but there is no study on the determination of secondary metabolites and genetic mechanism of *D. nobile* under different epiphytic patterns (Li et al., 2017; Wen et al., 2017). Using the combined analysis of transcriptomics and metabolomics, this study compared the differential genes and metabolites of *D. nobile* under the four epiphytic patterns of Danxia stone, Crushed stone, Sawdust, and Stump. Then, association analysis was carried out to explore the genetic mechanism of differences. The results were expected to provide reference for the planting of *D. nobile.*

## Materials and Methods

### Materials

*Dendrobium nobile* with the same genotype cultivated by Chishui Xintian Company (105°74′E, 28°56′N) was sampled for the following study. In 2018, capsules were collected from *D. nobile* plants in Dendrobium resource Park of Chishui Xintian company. After surface disinfection, callus and seedlings were induced by tissue culture, and then, the seedlings were cultured and planted to form full-grown plants. Notably, 1 g callus, 1 g seedlings, and 1 g rhizomes and leaves of *D. nobile* were randomly selected, each with three replicates. Total RNA was extracted by the Trozil method, and mixed in equal amounts for RNA-Seq sequencing to construct the nucleotide library of the whole organ and tissues of *D. nobile* during the whole growth period.

From the Dendrobium resource Park of Chishui Xintian Company, the 2-year-old stems with the same shape and size on Danxia stone (CSXTDS), Crushed stone (CSXTSS), Sawdust (CSXTJM), and Stump (CSXTSZ) were selected, respectively. Each stem was divided into two parts, and after triturating with liquid nitrogen, 200 mg of each stem was used to extract total RNA. One part was used for Illumina sequencing, and the other part was used to separate secondary metabolites for metabolome sequencing. Each sample had three biological replicates.

### Construction of cDNA Library and RNA-Seq Sequencing

Advanced molecular biology equipment should be used to detect the purity, concentration, and integrity of RNA samples after mixing. After the samples pass the test, the full-length cDNA of mRNA was synthesized by SMARTer™ PCR cDNA Synthesis Kit, and was then amplified by PCR. The end of the full-length cDNA was repaired, connected with SMRT dumbbell connector, digested by exonuclease, and then the sequencing library was obtained. After being inspected in the library, the full-length transcriptome was sequenced by PacBio instrument according to the target off-line data. The sequence was divided into full-length sequence and non-full-length sequence according to whether 3 “primer, 5” primer, and PolyA (optional) were present or absent. The full-length sequences from the same transcript should be clustered, high-quality sequences should be extracted, and then the transcript sequence after removing redundancy should be obtained. The nucleotide database of the whole growth period, whole tissues and organs, and the full length of the transcript of *D. nobile* was established.

### Illumina Sequencing and Differential Gene Screening

After the total RNA of the samples under different epiphytic patterns passed the quality inspection, the eukaryotic mRNA was enriched using the magnetic beads connected with Oligo(dT). The extracted mRNA is randomly fragmented into short fragments by Fragmentation Buffer, and a single-strand cDNA is synthesized by using the fragmented mRNA as a template with six-base random primers (Random hexamers). Buffer, dNTPs, RNaseH, and DNA polymerase I were then added for double-stranded cDNA synthesis. The second strand of cDNA was amplified by PCR to obtain the final sequencing library. The library was sequenced by Illumina Nova seq™ 6000 after passing the quality inspection, and the sequencing read length was paired-end 2 × 150 bp (PE150). *De novo* assembly of the transcriptome was performed with Trinity 2.4.0. Trinity groups transcript into clusters based on shared sequence content. Such a cluster of transcripts is very commonly referred to as a “gene.” The longest transcript in the cluster was chosen as the “gene” sequence (aka Unigene).

### Unigene Annotation and Functional Classification

Salmon was used to perform expression level analysis for Unigenes by calculating transcripts per million (TPM). The differentially expressed Unigenes were selected with log2 (fold change) > 1 or log2 (fold change) < −1 and with statistical significance (*p* < 0.05) by R package edgeR. All assembled Unigenes were aligned against the non-redundant (Nr) protein database Gene ontology (GO), SwissProt, Kyoto Encyclopedia of Genes and Genomes (KEGG), and eggnog databases using DIAMOND with a threshold *E* value < 0.00001. Pfam.

### Metabolome Sequencing and Secondary Metabolite Isolation

To determine the metabolites that significantly changed in *D. nobile* under different epiphytic patterns, four stem strips under four attachment methods, Danxia stone (CSXTDS), Crushed stone (CSXTSS), Sawdust (CSXTJM), and Stump (CSXTSZ) were analyzed in three replicates per group. The collected samples were thawed on ice, and metabolites were extracted from 20 μl of each sample using 120 μl of precooled 50% methanol buffer. Then, the mixture of metabolites was vortexed for 1 min and incubated for 10 min at room temperature, and stored at −20°C overnight. The mixture was centrifugated at 4,000 *g* for 20 min; subsequently, the supernatant was transferred to 96-well plates. Pooled quality control (QC) sample was also prepared by combining 10 μl of each extraction mixture. All samples were analyzed using a Triple TOF 5600 Plus higher solution tandem mass spectrometer (SCIEX, Warrington, United Kingdom). Chromatographic separation was performed using an ultra-performance liquid chromatography (UPLC) system (SCIEX, United Kingdom). During the entire acquisition period, the mass accuracy was calibrated every 20 samples. Furthermore, a QC sample was analyzed every 10 samples to evaluate the stability of the LC-MS.

The group datasets were normalized before the analysis was performed. Data normalization was performed on all samples using the probabilistic quotient normalization algorithm. Then, QC-robust spline batch correction was performed using QC samples. The *p* value analyzed by Student’s *t*-test, which was then adjusted for multiple tests using an FDR (Benjamini–Hochberg), was used for the selection of different metabolites. We also conducted the supervised PLS-DA using metaX for variables that discriminate profiling statistical method to identify more specific differences between the groups. The VIP cut-off value of 1.0 was set to select important features.

### Combined Analysis of Differential Genes and Differential Metabolites

Based on the RNA-Seq sequencing, differential analysis of gene expression was performed. Differential genes were screened with *p* < 0.05, FC > 2, or < −2 as criteria, and statistical analyses such as fold-change and *t*-test were used for univariate analysis, and q-values were obtained by BH correction. Combined with multivariate statistical analysis of the VIP (Variable Important for the Projection) value obtained by PLS-DA, the satisfying ratio ≥ 2 or ratio ≤ 1/2, *p* value ≤ 0.05, and VIP ≥ 1 of differential metabolites were screened. R language was used to standardize the expression values of differential genes and differential metabolites, and correlation analysis was performed. Taking *p* < 0.05, *r* > 0.8 as the standard, the KEGG pathway annotation was used to analyze the intersection in order to obtain key genes and metabolites.

### Quantitative Real-Time PCR Validation

To verify the accuracy of transcriptome data, differential genes (DEG) were selected for qRT-PCR verification. The stems of 2-year-old *D. nobile* collected on the Danxia stone (CSXTDS), Crushed stone (CSXTSS), Sawdust (CSXTJM), and Stump (CSXTSZ) were used as materials. Total RNA was extracted by BIOFIT polysaccharide polyphenol biological kit, and cDNA was synthesized by TIANGEN reverse transcription kit. The volume of qRT-PCR reaction system was 10 μl in total, containing 1 μl cDNA template with a concentration of 1–10 ng/μl, 5 μl SYBR Green qPCR Master Mix (Universal), 0.2 μl primer each with a concentration of 10 μmol/L, and 3.6 μl ddH_2_O. The reaction was performed using the following conditions: denaturation at 95°C for 30 s, followed by 40 cycles of amplification (95°C for 10 s, 60°C for 30 s, and 72°C for 30 s), and extension at 95°C for 15 s. For each sample, three technical replicates of the qRT-PCR assay were used with three biological replicates. Gene expression was evaluated using the 2^–ΔΔCt^ method.

## Results

### Analysis of Illumina Sequencing Results

**FIGURE 1:**
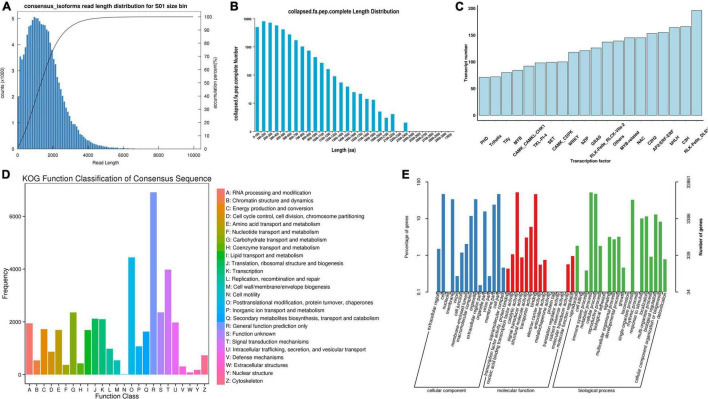
Sequencing results of third-generation transcriptome. **(A)** Length distribution of transcripts after redundancy removal; **(B)** Length distribution of complete ORF; **(C)** Statistics of different types of transcription factors; **(D)** KOG function annotation; and **(E)** GO function annotation.

Based on the Illumina sequencing, 370,032 CCS polished sequences were obtained. After clustering the full-length nonchimeric sequences, 118,574 high-quality consistent sequences were obtained. Then, after removing redundancy, 68,401 transcript sequences were obtained (Figure 1A). The length of the sequence was less than 6,000 nt, mainly distributed around 2,000 nt. With the increase of dress matching length, the single gene showed a decreasing trend. By analyzing the transcript sequence, 2,034 alternative splices were obtained for each sample, and a total of 25,591 SSRs and 9,427 lncRNA were predicted. In the prediction of coding sequence (CDs), 36,730 regions of CDs were obtained, and the complete open reading frame (ORF) length was mainly distributed in 100–200 aa, accounting for 21.45% (Figure 1B). A total of 5,572 transcription factors were predicted (Figure 1C), which were distributed in 4,065 gene families. To obtain the annotation information of transcripts, the obtained transcripts were compared with NR, Swissprot, GO, COG, KOG, Pfam, and KEGG databases.

A total of 59,148 transcripts were annotated, and 59,065, 55,554, 40,735, and 44,748, 26,738, and 36,610 transcripts were annotated in NR, eggNOG, Swissprot, and Pfam, KEGG, KOG databases, respectively (Figure 1D). All these databases divided transcripts into 25 categories, among which the functional annotation of KOG database was relatively comprehensive. Most life activities were included in the annotation, and the number of genes related to general function prediction was the largest, with 6,021. In contrast, the number of genes associated with cell motility was only 15, and other kinds of transcripts were expressed differently. Among them, the number of genes related to metabolic function was 1,517. GO database annotated 33,861 transcripts (Figure 1E), which were mainly divided into three aspects: biological process, molecular function, and cellular component. Among them, Oxidation-reduction process (2,715 genes), ATP binding (4,148 genes), and Integral component of membrane (7,011 genes) were the most abundant subgroups, respectively. This sequencing constructed a nucleotide database of the whole growth period, all tissues and organs, and full-length transcripts of *D. nobile*.

### Analysis of RNA-Seq Sequencing Results Under Different Epiphytic Patterns

**FIGURE 2:**
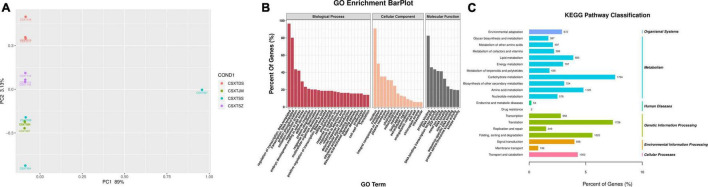
Transcriptome sequencing results. **(A)** PCA map of sample differential genes; **(B)** GO functional annotation map of genes; and **(C)** KEGG transcriptome annotation results.

To obtain the gene expression of *D. nobile*, the RNA of *D. nobile* was extracted from four different epiphytic patterns, and then, a sequencing library was established to sequence the qualified samples. The PCA diagram shows (Figure 2A) that the sample has a high resolution and can be used in subsequent quantitative and qualitative studies. The 12 sample libraries constructed were sequenced on a machine and were tested and then qualified (Table 1), resulting in a total of 165,609 transcripts being spliced into 80,135 Unigenes. Different authoritative databases, namely, NCBI_nr, GO, KEGG, Pfam, Swiss-Prot, and eggNOG were selected for a comparison of Unigenes.

**TABLE 1:** Transcriptome sequencing results.

Samples	Raw_Reads	Valid_Reads	Valid%	Q20%	Q30%	GC%
CSXT013	49,669,070	48,255,252	97.15	98.25	94.43	47.14
CSXT017	57,695,118	56,023,998	97.1	98.26	94.43	46.89
CSXT018	45,646,752	44,231,148	96.9	98.35	94.67	46.95
CSXT084	46,658,872	45,367,348	97.23	98.24	94.37	45.85
CSXT086	45,166,582	42,998,662	95.2	98.32	94.59	46.5
CSXT087	39,684,332	38,740,132	97.62	98.3	94.54	46.38
CSXT024	49,847,190	48,614,568	97.53	98.34	94.63	46.68
CSXT027	42,795,584	41,971,920	98.08	98.3	94.47	44.17
CSXT028	49,252,856	48,219,690	97.9	98.25	94.38	46.6
CSXT115	44,594,872	43,740,732	98.08	98.25	94.39	46.23
CSXT116	52,407,542	51,185,366	97.67	98.19	94.23	46.15
CSXT118	53,060,766	51,965,238	97.94	98.21	94.31	46.53

The GO (Gene Ontology) functional annotation results showed (Figure 2B) that a total of 27,761 (34.64%) Unigenes were annotated in three areas, namely, molecular functions, cellular components, and biological processes. In terms of molecular function, it was mainly concentrated in protein binding, molecular function, and ATP binding. In terms of cellular components, it was mainly enriched in three aspects, namely, nucleus, cytoplasm, and plasma membrane. In terms of biological process, it was mainly enriched in three aspects: biological process, transcriptional regulation, and transcription. The KEGG results showed (Figure 2C) that a total of 23,454 Unigenes were annotated, which were related to 141 different metabolic pathways. These pathways were mainly divided into six categories (i.e., Organismal Systems, Metabolism, Human Diseases, Genetic Information processing, Environmental Information Processing, and Cellular Processes). Interestingly, Metabolism, Genetic Information Processing, and Environmental Information Processing were the three main genes. Carbohydrate metabolism (1,794 genes), Translation (1,726 genes) and folding, sorting, and degradation (1,325 genes) were the three main categories. These important metabolic pathways may affect the synthesis of active components in *D. nobile*. In addition, a total of 32, 98, 89, 115, and 19 genes were related to Indole alkaloid biosynthesis (K01593, K21026), Isoquinoline alkaloid biosynthesis (K01593, K00811, K00276, K00422, K14454, K00815, K15849, K21311, K14455), Tropane, piperidine, and pyridine alkaloid biosynthesis (K00811, K00276, K14454, K08081, K00815, K15849, K00817, K14455), Flavonoid biosynthesis (K13081, K01859, K00487, K08695, K13065, K00475, K00660, K05278, K05277, K05280, K13083, K13082, K00588, K09754), and Isoflavonoid biosynthesis (K13258, K13264, K13260), respectively. There remains a sea of unigenes that were found in the plant hormone signal transduction (524 unigenes), plant-pathogen interaction (527 unigenes), MAPK signaling pathway (414 unigenes), and Circadian rhythm (146 unigenes) categories. These pathways not only play a significant role in plant growth and development but were also related to the synthetic processes for primary and secondary products.

### Differential Gene Analysis Under Different Epiphytic Patterns

**FIGURE 3:**
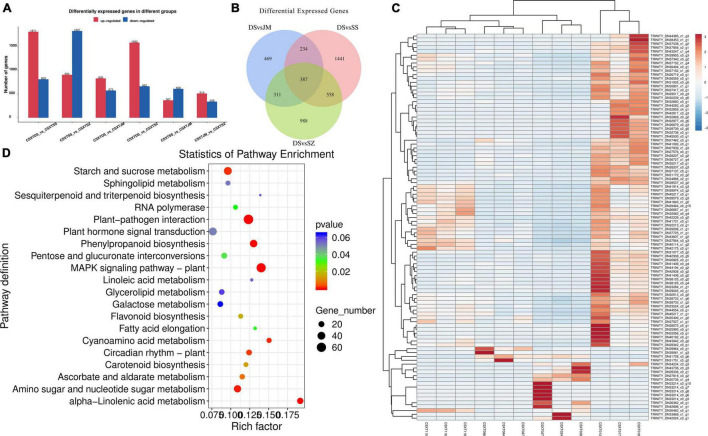
Statistics of differentially expressed genes. **(A)** Number statistics of differentially expressed genes; **(B)** Veen map of Danxia stone; **(C)** Differential gene clustering heat map; and **(D)** Enrichment of KEGG pathway of differential genes.

To explore the key differential genes of *D. nobile* under different epiphytic patterns, the genes of Danxia and other epiphytic patterns were compared and analyzed. The change in gene expression was different under each epiphytic pattern (Figures 3A,C), and the change in gene expression in Danxia stone was the largest. There were 1,401 differential genes in the DSvsJM comparison group; 2,620 differential genes in the DSvsSS comparison group and 2,244 differential genes in the DSvSZ comparison group. In the three comparison groups, 828, 1,811, and 1,582 genes were upregulated and 573, 809, and 662 genes were downregulated, respectively. Among them, there were 387 differentially shared genes in the three comparison groups (Figure 3B), and 289 genes were annotated. These genes encode different genes and proteins, including α-galactosidase-like, chalcone synthase, retrovirus-related pol polyprotein from transposon TNT 1-94, allene oxide synthase 1, mitogen-activated protein kinase kinase kinase ANP1-like, cinammate 4-hydroxylase, 3-ketoacyl-CoA synthase 4-like, E3 ubiquitin-protein ligase PUB23-like, fructokinase-1-type, linoleate 13S-lipoxygenase 2-1, chloroplastic-like, L-ascorbate oxidase homolog, flavonoid 3′-monooxygenase-like isoform X2, and WRKY transcription factor.

The KEGG analysis annotated differential genes in different pathways (Figure 3D), including starch and sucrose metabolism (dct00500), MAPK signaling pathway-plant (dct04016), carotenoid biosynthesis (dct00906, zma00906), ubiquitin mediated proteolysis (cre04120), fructose and mannose metabolism (dct00051), pentose and glucuronate interconversions (smo00040), proteasome (dct03050), flavonoid biosynthesis (nnu00941), plant-pathogen interaction (csat04626, han00564, dct04626), pyruvate metabolism (dct00620), glycerophospholipid metabolism (han00564), endocytosis (vvi04144), folate biosynthesis (ghi00790), amino sugar and nucleotide sugar metabolism (dct00520), carbon fixation in photosynthetic organisms (dct00710), alpha-Linolenic acid metabolism (han00592), and other pathways. These genes were selected for further correlation analysis.

### Analysis of Differential Metabolites Under Different Epiphytic Patterns

**FIGURE 4:**
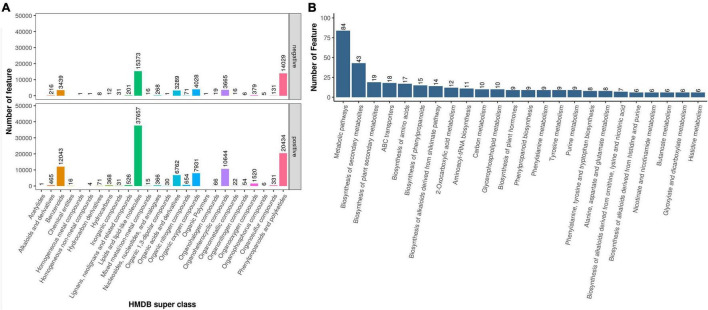
Metabolome sequencing results. **(A)** Metabolite HMDB Super class classification map and **(B)** Metabolite KEGG pathway enrichment.

To explore the metabolic differences of *D. nobile* under different epiphytic patterns, the samples were analyzed by metabolic analysis. A total of 29,518 metabolites were identified. The results of HMDB superclass classification showed (Figure 4A) that lipid molecules were the most abundant metabolites, followed by phenylpropane and polyketones, and then heterocyclic compounds, organic oxygen compounds, organic acids, and their derivatives. Results of KEGG database showed (Figure 4B) that differential metabolites were mainly enriched in metabolic process, secondary metabolite biosynthetic process, ABC transporter, cyanoamino acid metabolism, and phenylpropanoid biosynthesis.

**FIGURE 5:**
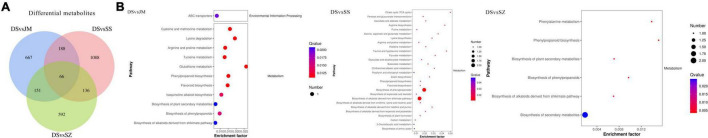
Statistics of differential metabolites. **(A)** Veen plot of differential metabolites and **(B)** KEGG enrichment pathway of differential metabolites.

Comparing Danxia stone with the other three epiphytic patterns, the results of analysis showed (Figure 5A) that there were 1,072 differential metabolites and 39 secondary metabolites in the DSvsJM comparison group. There were 1,478 differential metabolites, and 39 metabolites were annotated in DSvsSS comparison group. There were 945 differential metabolites, and 21 secondary metabolites were annotated in the DSvsSZ comparison group. In the three comparison groups, 407, 535, and 343 metabolites were upregulated, and 665, 943, and 602 were downregulated. There were 66 differential metabolites in common among the three comparison groups, including butadiene-styrene rubber, 2-methyl-4-heptanone, (Z)-3-nonen-1-ol, 3,5,5-trimethylhexanal, *S*-propyl-L-cysteine, 2-pentadecylfuran, and other metabolites. KEGG pathway (Figure 5B) showed that the differential metabolites of the DSvsJM, DSvsSS, and DSvsSZ groups were enriched in 14, 31, and 7 pathways, respectively. The common pathways of different metabolites in the three comparison groups were phenylpropanoid biosynthesis, biosynthesis of alkaloids derived from shikimate pathway, biosynthesis of plant secondary metabolites, biosynthesis of secondary metabolites, metabolic pathways, flavonoid biosynthesis, and arginine and proline metabolism. An analysis showed that the Crushed stone and Danxia stone had the most differential metabolites, and Danxia stone had an effect on the synthesis of phenyl propionic acids, alkaloids, flavonoids and other amino acids, and secondary metabolites. These differential metabolites were selected for further experiments.

### Combined Analysis of Differential Genes and Metabolites

To understand the connection between genes and metabolites under different epiphytic patterns, the KEGG pathway was analyzed, and the correlation analysis of differential genes and differential metabolites was performed. There were 387 differential genes among the three groups of samples, corresponding to 66 differential metabolites. We detected 542 significant associations (*p* < 0.01, *R* > 0.8) between 386 genes and 66 metabolites. Among them, different metabolites are associated with many different genes. Dimethomorph was associated with the maximum number of genes (89), and many metabolites were only associated with *RVE1*, such as Epicatechin-(4beta- > 8)-ent-epicatechin, Procyanidin_B4, Procyanidin_B2, Procyanidin_B5, and dTDP-4-amino-4,6-dideoxy-5-C-methyl-D-allose. Among them, *ACS1* was negatively correlated with hydroxymethylbilane, which indicated that this gene had an inhibitory effect on the synthesis and accumulation of hydroxymethylbilane. There was a positive correlation between *LOX2.3* and 2,3-Dinor-8-iso_prostaglandin_F1alpha, indicating that this gene can promote the synthesis and accumulation of lipid molecules and lipid-like molecules.

**FIGURE 6:**
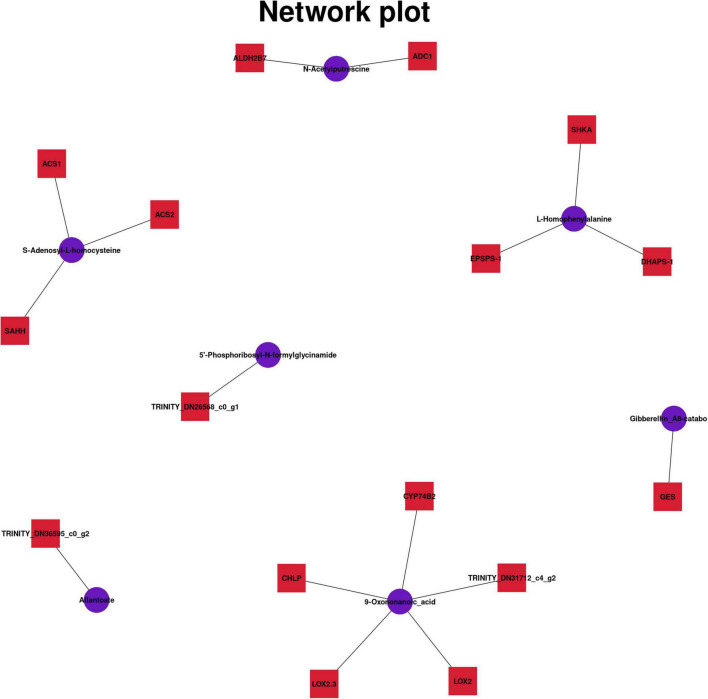
Related genes and metabolites in six pathways.

Different metabolites were associated with different genes (Figure 6), and six common pathways of KEGG pathway were observed. *CYP75B2* and Malonylapiin are coenriched in the Flavone and flavonol biosynthesis (map00944) pathway, and both showed a downward trend. *CYP75B2* encodes flavonoid 3′-monooxygenase, which was also associated with cell membrane (GO:0016020) and oxidoreductase activity (GO:0016709) and the synthesis process of secondary metabolites (GO:0044550), which affected not only the metabolism of flavonoids, but also the metabolism of other secondary products (Zhang X. et al., 2020), such as anthocyanin synthesis (Jin et al., 2020). *ALDH2B7, ADC1*, and N-Acetyl putrescine were enriched in the metabolic pathway of arginine and proline. *EPSPS-1, SHKA, DHAPS-1*, and L-Homophenylalanine were enriched in the metabolic pathway of phenylalanine. These genes regulated the anabolism of amino acid analogs. *TRINITY_DN29493_c2_g1* was related to the synthesis of 5′-Phosphoribosyl-N-formylglycinamide, and *TRINITY_DN36595_c0_g2* was related to the synthesis of Allantoate. They were jointly enriched in the metabolism pathway of purine, and the gene expression was upregulated, whereas the metabolite expression was downregulated, indicating that this gene may inhibit purine metabolism. *GES* and Gibberellin_A8-catabolite were coenriched in the diterpenoid biosynthesis. *ACS1, SAHH, ACS2*, and *S*-Adenosyl-L-homocysteine were coenriched in the cysteine and methionine metabolism (map00270) pathway. The expression of *ACS1* and *ACS2* was upregulated and the expression of *SAHH* was downregulated, which jointly regulated cystine and methionine metabolism. *CHLP, LOX2, LOX2.3, CYP74B2*, and *TRINITY_DN31712_c4_g2* were related to alpha-Linolenic acid metabolism (map00592). These genes were downregulated, indicating that the genes inhibited synthesis and accumulation of alpha-linolenic acid. The results showed that the metabolism of flavonoids, purines, and alpha-linolenic acid in Danxia stone decreased to varying degrees. There were a variety of regulatory mechanisms in organisms, and genes and metabolites had different regulatory modes.

### Expression Characteristics of Differential Metabolites and Genes

**FIGURE 7:**
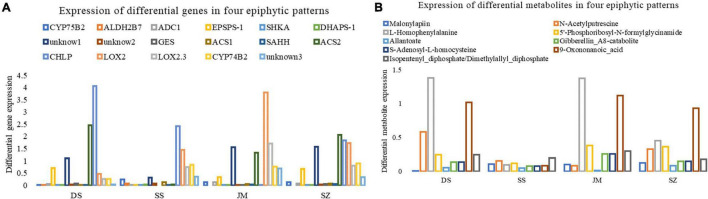
Changes of differential genes and differential metabolites under different epiphytic patterns. The abscissa represents four different epiphytic patterns. Danxia stone (CSXTDS, DS), Crushed stone (CSXTSS, SS), Sawdust (CSXTJM, SS), and Stump (CSXTSZ, SZ). **(A)** Differential gene expression. Unknow1 is *TRINITY_DN29493_c2_g1*, unknow2 is *TRINITY_DN36595_c0_g2*, unknow3 is *TRINITY_DN31712_c4_g2.* **(B)** Peak areas of differential metabolites under different epigenetic patterns.

According to the KEGG pathway enrichment of differential genes and metabolites, 17 differential genes (Figure 7A) and eight metabolites (Figure 7B) were finally obtained. After the expression and metabolite peak area of genes were standardized in R, their expression characteristics were analyzed. The results showed that the expression of *CYP75B2* and the metabolite Malonylapiin was downregulated in Danxia stone, while the expression levels were higher in other epiphyte modalities, indicating that the biosynthesis of flavonoids and flavonols was reduced in Danxia stone. In the stone epiphytic patterns, *CHLP* was most actively expressed, encoding geranylgeranyl reductase, affecting the synthesis of metabolites Isopentenyl-diphosphate and geranylgeranyl-diphosphate, and regulating the terpenoid backbone biosynthesis. The biosynthesis of terpenoid backbone was not active in the wooden forms, and the most active one was *LOX2*, encoding lipoxygenase, but its expression level was low in the epilithic forms. The content of the corresponding metabolites in the wooden forms was higher than that in the stone patterns. It indicated that differential regulation of alpha-linolenic acid metabolic pathways occurs in the two epiphytic modalities. *TRINITY_DN31712_c4_g2* also encoded lipoxygenase, but its expression was downregulated, indicating that lipoxygenase was regulated by different genes and was a key enzyme in the alpha-linolenic acid metabolic pathway. L-Homophenylalanine was higher in both Danxia stone and Stump, although almost absent in Crushed stone, which indicated that amino acid metabolism was more active in Danxia and Sawdust. In general, the content of secondary metabolites was higher in Danxia stone than in other epiphytic patterns, and lower in Crushed stone.

### qRT-PCR Validation

**FIGURE 8:**
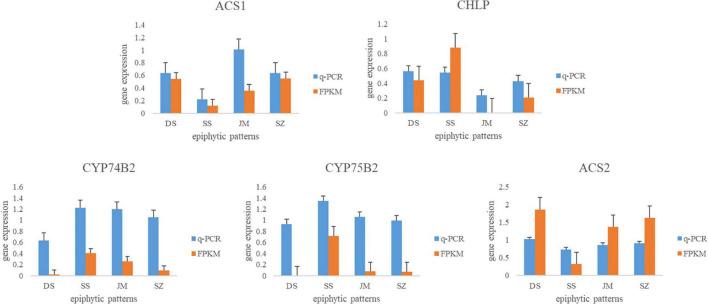
qRT-PCR analysis of differential genes in *Dendrobium nobile*. The ordinate corresponds to the average change of expression value, the abscissa represents four different epiphytic patterns, and the FPKM represents the value of transcriptome sequencing.

To validate the reliability of the transcriptome data, we selected five DEGs and analyzed their expression levels by qRT-PCR. The actin gene of *D. nobile* was used as a reference control. Primers were designed using Primers Blast on NCBI website (Table 2). The results showed that the expression patterns of the five DEGs were consistent with the transcriptome sequencing data (Figure 8). In Danxia stone, the expression of *CYP75B2* was downregulated, and in the Crushed stone, the expression of *CHLP* was the most active, which confirmed the reliability of our transcriptome data.

**TABLE 2:** The information of qPCR primers.

Gene name	Primer	Tm	Length	Product length
CHLP-F	ACGTCTCACCTGATTTCTACGG	59.84	22	85
CHLP-R	TCTGATTTGTGGGTGACGGTG	60.54	21	
ACS1-F	TGGTTTAGGGTTTGCTTCGC	59.04	20	148
ACS2-R	TCAACTTCAGCCCAGTGTTCT	59.51	21	
CYP75B2-F	GTGGGGAGGAGAGTGTTTGG	60.0	20	116
CYP75B3-R	CCAAGCCCAGGCACAAAATC	60.0	20	
ADC1-F	GGATGTCCTGCGAGCGATG	60.95	19	82
ADC2-R	ACTGTGAGCGTATTCCTCGG	59.54	20	
CYP74B2-F	GCTAGGCTTCAACGCATTCG	60.0	20	92
CYP74B3-R	GTCTCTGCTTCAGCCCTGTT	60.0	20	
ACS2-F	GTGAAGCTTAACGTGTCGCC	59.8	20	115
ACS3-R	TTATTCTCCTCAGCGCCACC	59.8	20	
actin-F	AATCCCAAGGCAAACAGA	51.0	18	–
actin-R	CACCATCACCAGAATCCAG	53.0	19	

## Discussion

In this study, through transcriptomic and metabolic analysis, the hub genes and pathways generated by *D. nobile* under different epiphytic patterns were identified, and the genetic mechanism of the differences was gained. In this study, we screened the differential genes related to differential metabolites. *CYP75B2*, which encoded flavonoid 3′-monooxygenase, regulated the metabolism of Malonylapiin, *ACS1* and *ACS2* regulated the synthesis of *S*-Adenosyl-L-homocysteine, and indirectly regulated the metabolism of cysteine and methionine, and *CHLP* and *CYP74B2* inhibited the synthesis and accumulation of α-linolenic acid. The amino acid metabolism in Danxia stone was increased, and it was closely related to *EPSPS-1, SHKA, DHAPS-1*, *ACS1*, *SAHH*, *ALDH2B7*, and *ADC1*. Amino acids, as signal molecules, were involved in regulating the synthesis of ethylene and other hormones in plants (Wu et al., 2020), and had a certain impact on the tissue metabolism, growth, maintenance, and repair of *D. nobile* (Heinemann and Hildebrandt, 2021). Danxia stone may induce certain stress response and interfere with the metabolism of amino acids, which was the result of *D. nobile* adaptation to the environment (Joshi et al., 2010). Danxia stone may be different from other epiphytes due to different contents of mineral elements and trace elements, and the response concentration and trend of different plants to various trace elements are different (Liu et al., 2010). Mineral nutrition may indirectly affect the synthesis and accumulation of flavonoids, terpenes, and other components in plants by affecting the levels of endogenous hormones in plants. In general, the content of total metabolites in Danxia stone was higher, which indicated that under the cultivation method of Danxia stone, the content of medicinal active ingredients was higher, and the quality of medicinal materials was good.

In this study, the full-length transcriptome database of the whole organs and tissues of *D. nobile* during the whole growth period was constructed by sequencing, which provides a basis for other studies of this species. Through the integrated omics analysis of transcriptome and metabolome, this study obtained the main metabolic pathways and genes produced by differences, and had a certain understanding of genetic mechanism concerning the epiphytic patterns of orchid plants. Theoretically, the cultivation method of Danxia was more conducive to the accumulation of metabolites in *D. nobile*, which was suitable for field cultivation. However, the accumulation of secondary metabolites is related not only to the cultivation mode, but also to the root fungi, bacteria, light, and harvesting season, which needs further research. Besides, this study could be serving as a high-quality reference for subsequent genomics and bioinformatics research of additional species within this genus.

## Conclusion

In this study, based on the PacBio SMRT sequencing and Illumina sequencing, a full-length transcriptome database of *D. nobile* during the whole growth period and whole organ tissue was constructed. UPLC-mass spectrometry was used to study the changes in metabolites of *D. nobile* under different epiphytic patterns. Combined transcriptome and metabolome analysis were used to explore the differences and genetic mechanism of *D. nobile* under different epiphytic patterns.

As a result, a total of 80,135 Unigenes and 29,518 metabolites were obtained. Among them, there were 387 differential genes, corresponding to 66 metabolites, of which 542 had significant correlations. The metabolic pathways which have changed were mainly flavone and flavonol biosynthesis, arginine and proline metabolism, metabolic pathway of phenylalanine, purine metabolism, cysteine and methionine metabolism, and alpha-linolenic acid metabolic pathway. Differential metabolites included Malonylapiin, *N*-Acetylputrescine, L-Homophenylalanine, 5′-Phosphoribosyl-N-formylglycinamide, Allantoate, Gibberellin_A8-catabolite, *S*-Adenosyl-L-homocysteine, 9-Oxononanoic_acid, Isopentenyl_diphosphate, and Dimethylallyl_diphosphate. Relevant regulatory genes included *ALDH2B7, ADC*, *EPSPS-1, SHKA*, *DHAPS-1*, *GES, ACS1, SAHH, ACS2*, *CHLP, LOX2, LOX2*.*3*, and *CYP74B2*. Among them, *ALDH2B7, ADC1* were related to arginine and proline metabolism, *EPSPS-1, SHKA, DHAPS-1* were related to metabolic pathway of phenylalanine, and *ACS1, SAHH, ACS2* were related to cysteine and methionine metabolism. The unknown differential genes *TRINITY_DN29493_c0_g2, TRINITY_DN36595_c0_g2* were related to purine metabolism. *TRINITY_DN31712_c4_g2* were related to alpha-linolenic acid metabolic pathway, which regulated lipoxygenase with *LOX2* and *LOX2.3*. Multiple metabolic pathways included *CYP75B2*, *RVE1*, *ACS1*, *LOX2.3*, which jointly regulated the synthesis of metabolites. Different epiphytic patterns will affect the metabolic regulatory network of *D. nobile*, and the differences were mainly enriched in flavonoid metabolism, purine metabolism, terpenoid backbone biosynthesis, amino acid metabolism, and alpha-linolenic acid metabolism. The synthesis of flavonoids and the metabolism of purines in Danxia stone decreased, while the synthesis of terpenoids and metabolism of amino acids was increased. At the same time, the metabolite content in Danxia stone was higher, and the metabolite content in Crushed stone was lower, indicating that Danxia stone was conducive to the accumulation of secondary metabolites in *D. nobile*.

## Data Availability Statement

The datasets presented in this study can be found in online repositories. The names of the repository/repositories and accession number(s) can be found below: National Center for Biotechnology Information (NCBI) BioProject database under accession number PRJNA806713.

## Author Contributions

D-LX conceived, supervised, and wrote-reviewed the manuscript. QL and CL originally wrote and reviewed the draft. QL, CH, MW, TL, JL, JHS, and LL performed the experiments and carried out the analysis. JLS and D-LX designed the experiments. D-LX and YH cofounded and co-administrated the project. All authors approved the final version.

## Conflict of Interest

The authors declare that the research was conducted in the absence of any commercial or financial relationships that could be construed as a potential conflict of interest.

## Publisher’s Note

All claims expressed in this article are solely those of the authors and do not necessarily represent those of their affiliated organizations, or those of the publisher, the editors and the reviewers. Any product that may be evaluated in this article, or claim that may be made by its manufacturer, is not guaranteed or endorsed by the publisher.
